# Concerns with the Study on Australian and New Zealand Fish Oil Products by Nichols et al. (*Nutrients* 2016, *8*, 703)

**DOI:** 10.3390/nu9020137

**Published:** 2017-02-14

**Authors:** Benjamin B. Albert, José G. B. Derraik, Manohar L. Garg, David Cameron-Smith, Wayne S. Cutfield

**Affiliations:** 1 Liggins Institute, University of Auckland, Private Bag, Auckland 92019, New Zealand; j.derraik@auckland.ac.nz (J.G.B.D.); d.cameron-smith@auckland.ac.nz (D.C.-S.); w.cutfield@auckland.ac.nz (W.S.C.); 2 Nutraceuticals Research Group, School of Biomedical Sciences and Pharmacy, University of Newcastle, Callaghan NSW 2308, Australia; manohar.garg@newcastle.edu.au

We read with interest a study recently published in *Nutrients* by Nichols et al. [1], which reported that fish oil products available in Australasia are not oxidised and are accurately labelled for content. However, this study contains several issues, including apparent methodological flaws, an incomplete literature review, and undeclared conflicts of interest. Importantly, their own study has shown high levels of secondary oxidation in the surveyed products as per previous studies, even after accounting for flavouring ingredients.

First, the 10 products studied represent only a fraction of the more than 40 fish oil products available in Australasia, so that the study covered substantially fewer products than in other recent publications. This unexplained small sample size (even if chosen based on market share) suggests a possible selection bias, making the survey unrepresentative of the range of products available to consumers.

Second, the methodology used in the measurement of fatty acid concentration is insufficiently described for the study to be replicated. The authors stated in their article that “full details on analytical methods are available on request from ALS” [1], which was the company subcontracted to perform the analyses (ALS Food and Pharmaceutical). ALS have provided their method on condition of confidentiality, which limits the detail we can provide of our assessment of their method. Nevertheless, we find that it lacks description of key features necessary to determine fatty acid concentration in mg/g of oil, which is required in order to determine actual EPA and DHA content. If a quantitative method of fatty acid analysis was not used, these data should be retracted. Further, we note that the method of fatty acid analysis remains unavailable to the scientific community.

Third, Nichols et al. incorrectly characterise the methodology used in our own study [2] as non-standard. In our study, peroxide and anisidine values were determined in strict accordance with the methods of the European Pharmacopoeia [3]. Further, fatty acid content was determined using the quantitative method first described by Lepage and Roy [4]. This method is highly cited in the peer-reviewed literature (nearly 1600 Scopus citations) and widely used in studies involving fatty acid analysis of food fats, supplements, and biological fluids/tissues, published in some of the world’s top scientific journals (such as *New England Journal of Medicine, JAMA, Cell Metabolism, Proceedings of the National Academy of Sciences of the USA*, and the *American Journal of Clinical Nutrition*).

Fourth, Nichols et al. have supported their findings by citing personal communications and unpublished data, but they appear to have overlooked all of the independent studies from around the world showing under-delivery of *n*-3 PUFA content and high levels of oxidation in retail fish oil products. These studies indicate that 17% to 93% of fish oil products exceed recommended limits of primary oxidation at the time of purchase [5,6,7,8]. It is also important to clarify that the Ismail et al. review [9] does not include “over 2000 published analyses” showing low levels of oxidation in fish oil products as stated by Nichols et al. [1]; rather, the review in question only cites a figure based on unpublished data that have not been peer-reviewed. Furthermore, it is interesting that Nichols et al. observed a high rate of secondary oxidation in their own study (even after excluding flavoured products): the proportion of products exceeding the Global Organization for EPA and DHA Omega-3 (GOED) limit for anisidine value (3/8 or 37.5%) [1] was greater than in our study [2] and similar to a North American investigation [6].

In regards to labelled content, numerous studies have shown that under-delivery of *n*-3 PUFA content (i.e., <90% labelled) is common worldwide [7,8,10,11,12,13,14]. In addition, two previous Australian studies cited by Nichols et al. as evidence of accurate labelling of *n*-3 PUFA content did not describe methodology that could produce a quantitative analysis [15,16].

Lastly, it is baffling how the authors could have declared no conflicts of interest, when this is quite clearly not the case. Both Nichols and Sinclair are scientific advisors to the Omega-3 Centre, while Dogan is the Chair of the Omega-3 Centre and also a business director (APAC, Nutritional Lipids) of DSM Nutritional Products [17]. The latter is a multinational corporation that sells nutritional supplements (including fish oil), while the stated mission of the Omega-3 Centre includes “supporting the development of the market for (…) dietary supplements containing long chain omega-3s” [17]. Failure to declare these conflicts breaches international standards of transparency in publishing [18]. There is concern about conflict of interest and how it affects the scientific literature [19], with evidence that studies influenced by industry are biased towards showing favourable results [20].
